# Physiological response of *Pichia pastoris* GS115 to methanol-induced high level production of the Hepatitis B surface antigen: catabolic adaptation, stress responses, and autophagic processes

**DOI:** 10.1186/1475-2859-11-103

**Published:** 2012-08-08

**Authors:** Ana Leticia Vanz, Heinrich Lünsdorf, Ahmad Adnan, Manfred Nimtz, Chandrasekhar Gurramkonda, Navin Khanna, Ursula Rinas

**Affiliations:** 1 Technical Chemistry – Life Science, Leibniz University of Hannover, Hannover, Germany; 2 Helmholtz Centre for Infection Research, Inhoffenstrasse 7, Braunschweig, 38124, Germany; 3 Department of Chemistry, Government College University Lahore, Lahore, Pakistan; 4 International Centre for Genetic Engineering & Biotechnology, New Delhi, India

**Keywords:** *Pichia pastoris*, Proteome, *Aox1* promoter, Carbon metabolism, ER stress, Autophagy

## Abstract

**Background:**

*Pichia pastoris* is an established eukaryotic host for the production of recombinant proteins. Most often, protein production is under the control of the strong methanol-inducible *aox1* promoter. However, detailed information about the physiological alterations in *P. pastoris* accompanying the shift from growth on glycerol to methanol-induced protein production under industrial relevant conditions is missing. Here, we provide an analysis of the physiological response of *P. pastoris* GS115 to methanol-induced high-level production of the Hepatitis B virus surface antigen (HBsAg). High product titers and the retention of the protein in the endoplasmic reticulum (ER) are supposedly of major impact on the host physiology. For a more detailed understanding of the cellular response to methanol-induced HBsAg production, the time-dependent changes in the yeast proteome and ultrastructural cell morphology were analyzed during the production process.

**Results:**

The shift from growth on glycerol to growth and HBsAg production on methanol was accompanied by a drastic change in the yeast proteome. In particular, enzymes from the methanol dissimilation pathway started to dominate the proteome while enzymes from the methanol assimilation pathway, e.g. the transketolase DAS1, increased only moderately. The majority of methanol was metabolized via the energy generating dissimilatory pathway leading to a corresponding increase in mitochondrial size and numbers. The methanol-metabolism related generation of reactive oxygen species induced a pronounced oxidative stress response (e.g. strong increase of the peroxiredoxin PMP20). Moreover, the accumulation of HBsAg in the ER resulted in the induction of the unfolded protein response (e.g. strong increase of the ER-resident disulfide isomerase, PDI) and the ER associated degradation (ERAD) pathway (e.g. increase of two cytosolic chaperones and members of the AAA ATPase superfamily) indicating that potential degradation of HBsAg could proceed via the ERAD pathway and through the proteasome. However, the amount of HBsAg did not show any significant decline during the cultivation revealing its general protection from proteolytic degradation. During the methanol fed-batch phase, induction of vacuolar proteases (e.g. strong increase of APR1) and constitutive autophagic processes were observed. Vacuolar enclosures were mainly found around peroxisomes and not close to HBsAg deposits and, thus, were most likely provoked by peroxisomal components damaged by reactive oxygen species generated by methanol oxidation.

**Conclusions:**

In the methanol fed-batch phase *P. pastoris* is exposed to dual stress; stress resulting from methanol degradation and stress resulting from the production of the recombinant protein leading to the induction of oxidative stress and unfolded protein response pathways, respectively. Finally, the modest increase of methanol assimilatory enzymes compared to the strong increase of methanol dissimilatory enzymes suggests here a potential to increase methanol incorporation into biomass/product through metabolic enhancement of the methanol assimilatory pathway.

## Background

The methylotrophic yeast *Pichia pastoris* is nowadays a well-established eukaryotic host for the heterologous production of technical enzymes and also for protein pharmaceuticals [1,2]. *P. pastoris* naturally possesses two different genes (*aox1* and *aox2*) encoding alcohol oxidases for oxidation of methanol, the first step in methanol degradation [3]. *Aox1* is highly expressed during growth on methanol, thus the strong *aox1* promoter is utilized most often to drive the production of foreign proteins. Another attempt to enhance the yield of the target protein is to increase its chromosomal gene dosage which - up to a certain target-protein dependent extent - can further increase product levels [4-7]. However, increasing the gene copy number beyond a target protein-dependent threshold can have a severe impact on cell growth and viability including a reduced level of the target protein [5,8-10]. In particular, overburdening the cellular protein export machinery for production of extracellular proteins can cause severe stress responses [11]. The passage of export-destined proteins through the secretory pathway often presents a bottleneck leading in many cases to partial retention of the target protein in the endoplasmic reticulum (ER) and a transcriptional upregulation of the Unfolded Protein Response (UPR) and the ER-Associated Degradation (ERAD) pathway [12,13].

In this study we investigated the physiological changes accompanying the methanol-induced high-level production of HBsAg, the major surface antigen of the Hepatitis B virus (HBV) employed for vaccination against HBV, in *P. pastoris* under industrially relevant conditions. The antigen is a very stable and also a very hydrophobic protein and not secretable in yeast expression systems [14]. Thus, production occurs as intracellular protein in *P. pastoris* GS115 carrying 8 copies of the gene encoding mature HBsAg without employing yeast-derived secretory signals [4]. Cells are grown on defined medium in a controlled fed-batch procedure maintaining the methanol concentration at 6 g L^-1^ through continuous methanol feeding during the production phase [15]. Although not destined for export the HBsAg is translocated into the ER where it is retained leading to a bulging of the ER into cloud-shaped irregular formations [14]. The very hydrophobic HBsAg is not further passaged in the secretory pathway but assembles into well-ordered multi-layered lamellar structures in the ER which are transformable into virus-like particles (VLPs) during down-stream processing [14]. The high-level production and the retention of the protein in the ER are supposedly of major impact on the cell physiology. For a more detailed understanding of the cellular response to methanol-induced high level HBsAg production, we followed the changes in the yeast proteome and the ultrastructural cell morphology during the HBsAg production process.

## Results and discussion

The physiological alterations accompanying the adaption of recombinant *Pichia pastoris* to methanol-induced high-level production of HBsAg were followed by proteome analysis and electron microscopy. Cells were grown initially on glycerol, and after depletion of glycerol, methanol was added to a final concentration of 6 g L^-1^ to initiate HBsAg production. This methanol concentration was kept constant through appropriate methanol feeding for the remaining fed-batch phase of the cultivation [15]. After an initial growth arrest and a short period of adaptation to methanol (~3-4 h), cells started to consume methanol and the biomass increased from 60 to 100 g L^-1^ cell dry mass during the first 90–100 h of the methanol feeding phase [15]. During this period, the intracellular HBsAg levels increased strongly reaching a final maximum of 6–7 g L^-1^ (thereof 30% soluble HBsAg competent for assembly into VLPs). Beyond this period, cell viability declined concomitant with a decrease of the soluble fraction of HBsAg [15]. Thus, this production process is characterized by four distinct phases: *i*. glycerol batch phase, *ii*. adaptation phase to methanol, *iii*. production phase, and *iv*. decline phase. From all these phases representative samples were taken for proteome analysis. The complete list of all identified proteins with their corresponding changes and classified into functional categories and a 2D gel image indicating all identified proteins are found in the Additional file 1. Moreover, cell physiological changes were also followed by ultrastructural analysis using transmission electron microscopy.

### Carbon metabolism: cell adaptation to carbon source change from glycerol to methanol

**Figure 1:**
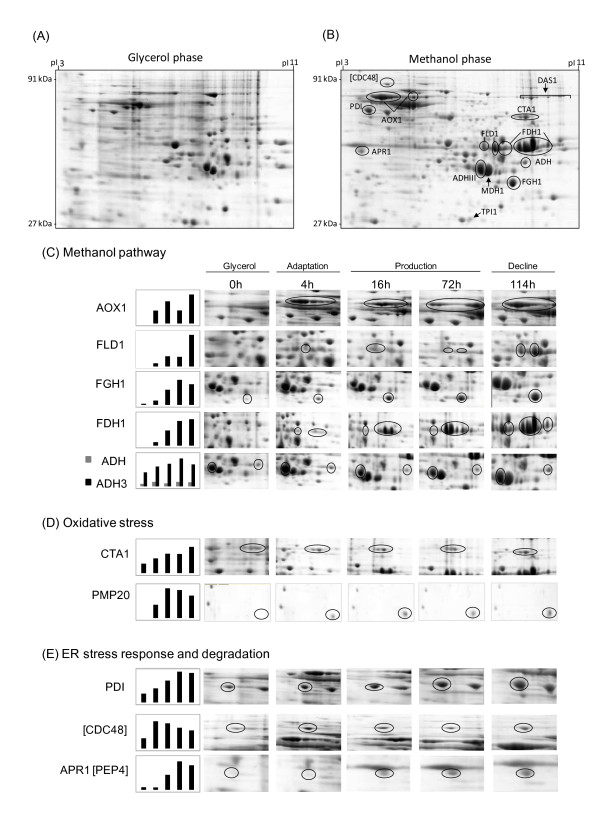
**Change of the intracellular proteome of *P. pastoris *GS115 in response to methanol-induced high-level production of the Hepatitis B surface antigen.** Sections of 2D gels representing parts of the intracellular proteome (**A**) at the end of the glycerol batch phase and (**B**) 114 hours after the onset of methanol feeding. For encircled protein spots (dark lines) time-dependent changes are given below. Magnified 2D gel sections from samples taken at the end of the glycerol batch phase and 4, 16, 72 and 114 hours after the onset of methanol feeding with representative proteins involved in (**C**) methanol metabolism and (**D**) oxidative and (**E**) ER stress responses (including also vacuolar degradation).

The intracellular proteome of *Pichia pastoris* exhibited a dramatic change in response to the shift from growth on glycerol to production and growth on methanol (Figure 1A and B).

#### Methanol metabolism

**Figure 2:**
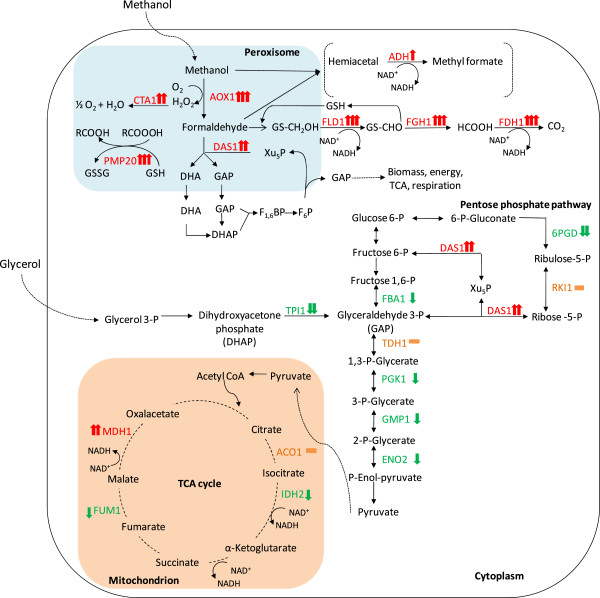
**Simplified carbon metabolic network.** Identified enzymes from pathways involved in glycerol and methanol metabolism, from the glycolytic and pentose phosphate pathway, and TCA cycle and their changes in response to the shift from growth on glycerol to growth and HBsAg production on methanol. The red arrow (↑) indicates an increasing and the green arrow (↓) a decreasing amount of the enzyme in the methanol fed-batch phase. An orange dash (−) indicates no significant change. One arrow indicates small (0.6-1 log2 change), two arrows strong (1–4 log2 change) and three arrows very strong changes (> 4 log2 change). Abbreviations (enzymes): AOX1, alcohol oxidase 1; CTA1, catalase; PMP20, peroxiredoxin; FLD1, formaldehyde dehydrogenase; FGH1, S-formylglutathione hydrolase; FDH1, NAD(+)-dependent formate dehydrogenase; ADH, alcohol dehydrogenase; DAS1, transketolase (dihydroxyacetone synthase); TPI1, triose phosphate isomerase; FBA1, fructose 1,6-bisphosphate aldolase; TDH1, glyceraldehyde-3-phosphate dehydrogenase; PGK1, 3-phosphoglycerate kinase; GMP1, tetrameric phosphoglycerate mutase; ENO2, enolase; 6PGD, 6-phosphogluconate dehydrogenase; RKI1, 1-ribose-5-phosphate ketol-isomerase; ACO1, aconitase; IDH2, subunit of mitochondrial NAD(+)-dependent isocitrate dehydrogenase; FUM1, fumarase; MDH1, mitochondrial malate dehydrogenase.

In particular, proteins required for methanol utilization which were only present in minor amounts during growth on glycerol started to dominate the proteome after the onset of the methanol feeding phase. Surprisingly, a major protein which accumulated during growth on methanol was identified as alcohol oxidase 1 (AOX1) (Figures 1A-C, 2 and Additional file 2). This HBsAg producing strain was first classified as Mut^s^[4], hence the presence of *aox1* and the accumulation of AOX1 unexpected. A comprehensive analysis verified the formation of AOX1 as well as the presence of the intact encoding gene *aox1* (for details please refer to Additional file 2). Thus, high level recombinant protein production may lead to impaired growth on methanol despite the presence of functional *aox1.*

In addition to AOX1 also the NAD^+^-dependent formate dehydrogenase (FDH1), the enzyme catalyzing the last step in the methanol dissimilation pathway increased to very high levels in the methanol feeding phase (Figures 1A-C and 2). The other two enzymes of the methanol dissimilation pathway, the S-(hydroxymethyl)-glutathione dehydrogenase (FLD1) and S-formylglutathione hydrolase (FGH1), also increased strongly but not as pronounced as AOX1 and FDH1 (Figures 1A-C and 2).

**Figure 3:**
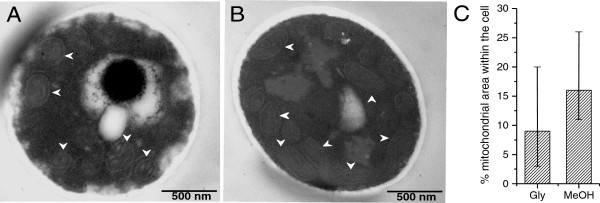
**Mitochondrial morphology and size changes during methanol-induced high-level production of the Hepatitis B surface antigen.** Transmission electron micrographs of *P. pastoris* GS115 (**A**) at the end of the glycerol batch phase and (**B**) 112 hours after the onset of methanol feeding. White arrow heads indicate mitochondria. (**C**) Quantification of the average total mitochondrial area within the cross sectional average cell area. The vertical bars encompass individual cell variance, e.g. the lowest and highest total mitochondrial area observed in a single cell in each growth phase; (left column) at the end of the glycerol batch phase and (right column) 112 hours after the onset of methanol feeding.

Interestingly, AOX1 already accumulated to high levels during the adapation phase to methanol (Phase II), while the subsequent enzymes of the methanol dissimilation pathway reached their highest concentrations in the production phase (Phase III) or even later in the decline phase (Phase IV) (Figure 1A-C). Methanol is not only oxidized by *Pichia pastoris* in the dissimilation pathway for potential energy generation through reoxidation of NADH in the respiratory chain but can also be incorporated into biomass in the assimilation pathway. In this case, formaldehyde, which is formed through oxidation of methanol by AOX1 (or AOX2), is not further converted to carbon dioxide, but condensed with xylulose 5-phosphate (Xu5P), through the action of dihydroxyacetone synthase (DAS1), a special transketolase which converts formaldehyde and Xu5P into the central C3-compounds dihydroxyacetone (DHA) and glyceraldehyde 3-phosphate (GAP) [16] (Figure 2). In contrast to the strong increase of the enzymes from the methanol dissimilation pathway, the level of DAS1 increased only moderately during the production phase (Figures 1A, B and 2) suggesting that the majority of methanol is processed through the dissimilation pathway leading to the generation of NADH and carbon dioxide. In fact, a carbon mass balance analysis revealed that 70 – 80% of the methanol metabolized is converted into carbon dioxide in the methanol fed-batch phase (data not shown). Interestingly, the mitochondrial area within cellular cross sections increased significantly during the methanol fed-batch phase (Figure 3) probably as a result of the elevated NADH supply from methanol dissimilation and the enhanced energy demand for growth, production and cell maintenance (e.g. repair and recycling).

#### *Glycolysis, pentose phosphate pathway, and tricarboxylic acid (TCA) cycle*

In contrast to the strong increase of the enzymes from the methanol utilizing pathways, in particular enzymes from the methanol dissimilation pathway, the enzymes from the glycolytic and the pentose phosphate pathway either decreased during the methanol fed-batch phase or did not show significant changes (Figure 2). A more pronounced decrease was observed for the triose phosphate isomerase (TPI1), the glycolytic enzyme required for channeling glycerol into the central carbon metabolic pathways (Figures 1A, B and 2 and Additional file 1). The enzymes of the TCA cycle did not show significant changes except for the mitochondrial malate dehydrogenase (MDH1) which increased strongly during the methanol feeding phase (Figures 1A, B and 2 and Additional file 1). MDH1 was already a prominent component of the intracellular proteome during growth on glycerol but increased even further in the methanol feeding phase (Figure 1A and B). Interestingly, the corresponding homolog in *A. niger* was also identified as major part of the intracellular proteome in well-aerated bioreactor cultures [17].

#### *Ethanol metabolism*

*P. pastoris* is generally classified as Crabtree-negative yeast and glycerol and methanol are not considered as fermentable carbon sources. However, two alcohol dehydrogenases (ADH and mitochondrial ADHIII) were identified in the proteome during growth on glycerol with ADHIII as a prominent component of the intracellular proteome (Figure 1A and C). The presence of alcohol dehydrogenases indicated the formation of ethanol in the glycerol batch phase which was confirmed by gas chromatography (1.3 g L^-1^ ethanol at the end of the glycerol batch phase). Previous reports also documented ethanol formation by *P. pastoris* during growth on excess glycerol [18,19]. Surprisingly, the amount of both enzymes increased even further in the methanol fed-batch phase (Figure 1A-C; ethanol concentration: 0.3 g L^-1^ after 112 hours of growth on methanol) indicating a potential involvement in metabolic activities beyond ethanol metabolism. In fact, it has been suggested that proteins of the ADH family from other methylotrophic yeasts (e.g. *C. boidinii*) are involved in fomaldehyde detoxification through formation of methyl formate [20]. A similar role may be also attributable to ADHs in *P. pastoris.*

### Stress responses

In the methanol fed-batch phase the cells are exposed to dual stress; stress resulting from methanol degradation and stress resulting from the production of the recombinant protein. The first, AOX catalyzed step during methanol utilization generates formaldehyde and hydrogen peroxide, both toxic compounds [16]. The delayed accumulation of formaldehyde processing enzymes compared to the rapid accumulation of AOX after the onset of methanol feeding (cf. preceding paragraph and Figure 1A-C) suggests a considerable negative impact of formaldehyde on the cells, in particular in the beginning of the methanol fed-batch phase.

#### *Oxidative stress response*

Methanol metabolism is mainly localized in the peroxisomes, membrane surrounded organelles which harbor the enzymes required for the initial steps of methanol metabolism (e.g. AOX, DAS1). During oxidation of methanol reactive oxygen species such as hydrogen peroxide but also peroxidated molecules are generated which need to be removed to prevent or minimize corresponding cell damage. At least two peroxisomal enzymes which are involved in the removal of reactive oxygen species, catalase (CTA1), which removes hydrogen peroxide [16,21,22], and a glutathione peroxidase or peroxiredoxin (PMP20), which removes peroxidated molecules, e.g. lipid hydroperoxides [16,22,23], increased strongly in the methanol fed-batch phase (Figures 1A, B, D and 2). CTA1 was already present in significant amounts during growth on glycerol and increased further in the adaptation and production phases (Figures 1A, B, D and 2). In contrast to CTA1, PMP20 was virtually absent during growth on glycerol but increased immediately in the methanol adaptation phase (Figures 1D and 2) indicating a more important role in methanol-related detoxification of reactive oxygen species. In fact, deletion of *pmp20* was more deleterious than deletion of *cta1* in methylotrophic yeast knockout strains (*C. boidinii* and *P. pastoris*) exposed to methanol [23,24]. It has been suggested that the presence of PMP20 is essential for maintaining peroxisomal membrane integrity during growth on methanol through removal of oxidized lipids [25]. Cyclophilin B, a cytoplasmic peptidyl-prolyl *cis-trans* isomerase (Cpr1), reported as environmental (oxidative)-stress responsive protein in *Saccharomyces cerevisiae*[26], also increased immediately in the methanol adaptation phase (Additional file 1) suggesting a stress-responsive function also for *P. pastoris.*

#### *Induction of UPR and ERAD pathway*

HBsAg is a very stable and also a very hydrophobic protein able to form VLPs during downstream processing [15]. The VLPs are further stabilized by intra- and intermolecular disulfide bonds [27]. During its methanol-induced high-level production HBsAg is translocated into the ER but not further processed in the secretory pathway [14]. The accumulation of HBsAg in the ER is leading to an expansion of the ER which bulges into cloud-shaped irregular formations. Thus, an induction of the UPR and a corresponding increase of ER resident chaperones or foldases would not be surprising. In fact, the amount of the UPR-inducible and ER-resident disulfide isomerase (PDI) increased strongly in the methanol fed-batch phase (Figure 1A, B, and E). Moreover, other UPR-induced proteins such as the mitochondrial chaperone SSC1 [28] also increased significantly during HBsAg production ( Additional file 1). In addition, two cytosolic chaperones and members of the AAA ATPase superfamily (ClpB = hsp104 and the AAA ATPase PAS_FragD_0026 = Cdc48) also revealed a strong increase in the methanol fed-batch phase (Figure 1A, B, E and Additional file 1) indicating an activation of the ERAD pathway. In *S. cerevisiae*, the homologs of both proteins have been identified as ER-stress responsive proteins, which participate in energy-driven disaggregation and degradation of ERAD substrates [29-31]. Hsp104 cooperates with hsp70 and hsp40 in disassembling protein aggregates for either proper refolding or degradation [29,31,32]. The AAA ATPase Cdc48 operates on the cytosolic part of the ER membrane actively involved in dragging misfolded proteins from the ER for subsequent degradation by the proteasome [33-35]. Here, their increase in response to ER stress was also verified for *P. pastoris*.

#### *Induction of other degradation pathways and autophagic processes*

**Figure 4:**
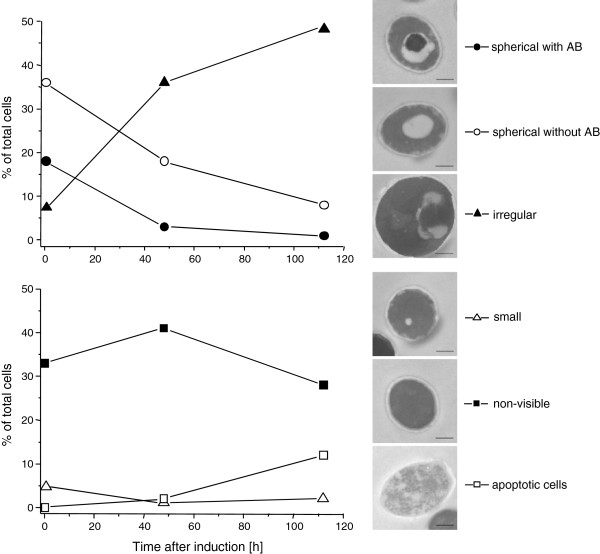
**Vacuole morphology changes in *P. pastoris *GS115 during methanol-induced high-level production of HBsAg.** Time-dependent change in the percentage of cells containing large spherical vacuoles with autophagic bodies (AB, ), cells containing large spherical vacuole without autophagic bodies (○), cells containing irregular vacuoles (▴), cells with small vacuoles (Δ), cells without any visible vacuoles (), and apoptotic cells (□). The bar in the electron micrographs of representative cells corresponds to 500 nm.

**Figure 5:**
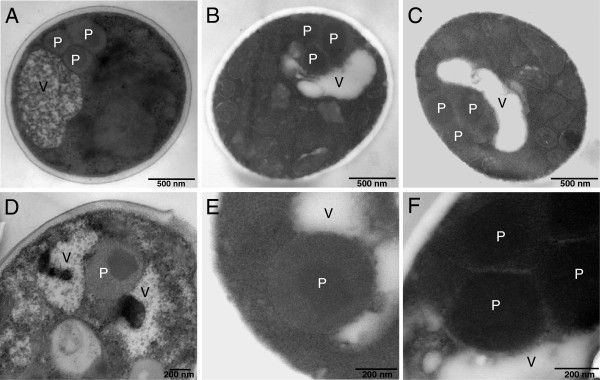
**Peroxisome sequestration via micropexophagy during methanol-induced high-level production of HBsAg.** (**A-C**) Representative transmission electron micrographs of ultrathin sectioned cells of *P. pastoris* GS115 growing for 112 hours on methanol. (**D-F**) Close-up views of vacuoles sequestering peroxisomes in cells growing for (**D**) 48 and (**E, F**) 112 hours on methanol. Abreviations: V, vacuole; P, peroxisome.

Moreover, a strong increase of the vacuolar aspartyl protease APR1 (PEP4 in *S. cerevisiae*) was observed in the methanol fed-batch phase (Figure 1A, B, E and Additional file 1) suggesting the induction of vacuolar degradation pathways in addition to ERAD. An electron microscopic examination of the cells revealed a drastic change in vacuole morphology after the start of the methanol fed-batch phase (Figure 4). At the end of the glycerol batch phase, the majority of cells possessed large spherical vacuoles of which many contained auto- phagic bodies. The appearance of spherical vacuoles with autophagic bodies indicate the onset of nutrient limiting conditions and the accompanying recycling of cell material [36]. After the start of methanol feeding, the number of cells with spherical vacuoles declined strongly and, instead, cells with irregularly shaped vacuoles increased in number (Figure 4). A closer electron microscopic examination revealed invagination of vacuoles (Figure 5) as is typically observed during peroxisome degradation by microautophagy (micropexophagy) [37,38]. Micropexophagy requires high levels of ATP [39] most likely available in the methanol fed-batch phase through primarily dissimilatory methanol catabolism. Interestingly, vacuolar enclosure was mainly related to peroxisomes (for details see Figure 5) and not to HBsAg deposits suggesting that vacuolar degradation pathways were not induced by HBsAg accumulation but most likely by damaged peroxisomes. In other cases, activation of autophagic processes have been reported in *S. cerevisiae* and mammalian cells upon induction of ER stress through addition of reducing agents [40] and tunicamycin and thapsigargin [41], respectively. Moreover, the analysis of the interactome of a degradation-prone and secreted Fab fragment in *P. pastoris* revealed mainly proteasomal degradation but also degradation via vacuolar pathways [42]. Also, analysis of the effects of producing folded-state stability variants of human lysozyme on the activation of stress responsive pathways revealed a reverse correlation of protein stability versus activation of degradative processes such as ERAD and ER-phagy, apparent through enhanced expression of e.g. *sec61* and e.g. *atg1*, respectively [12]. However, in our case invaginated vacuoles were either closely connected to peroxisomes (Figure 5) or otherwise did not show any clear connection to other organelles (e.g. mitochondria or ER). Autophagy of peroxisomes (pexophagy) has been previously reported for *P. pastoris* upon shifting from methanol to ethanol or glucose but not during growth on methanol. Our findings suggest that constitutive autophagic recycling of peroxisomes might be part of the house-keeping machinery of *Pichia pastoris* also under methanol growth conditions helping cells to deal with damage caused by reactive oxygen species created through methanol oxidation. Vacuolar enclosure of peroxisomes was already apparent in the middle of the production phase (Figures 4 and 5D) and increased further during the ongoing methanol fed-batch phase (Figures 4 and 5). Indeed, it has been shown for the methylotrophic yeast *Hansenula polymorpha* that constitutive pexophagy is vital during growth on methanol as mutant cells with defects in autophagy displayed reduced vitality [43] and damaged peroxisomes are rapidly subjected to autophagic degradation in *H. polymorpha*[44]. Thus, the strong increase in the vacuolar protease APR1 during the methanol fed-batch phase might not be related to HBsAg production but to vacuolar degradation of damaged peroxisomes.

## Conclusions

The shift from growth on glycerol to growth and production on methanol leads to a drastic change in the yeast proteome. In particular, enzymes from the methanol dissimilation pathway start to dominate the proteome while enzymes from the methanol assimilation pathway, e.g. the transketolase DAS1, only show a moderate increase during the production phase suggesting this pathway as a potential target for metabolic engineering to enhance methanol assimilation, e.g. though enhanced expression of genes from the methanol assimilatory pathway [e.g. [45]. Moreover, the strong increase of alcohol dehydrogenases, ADHs, in the methanol-fed batch phase indicate that ADHs may play an important role in formaldehyde detoxification in *P. pastoris.*

The accumulation of HBsAg in the ER of *P. pastoris* leads to the induction of the UPR and the ERAD pathway suggesting that potential degradation of HBsAg may proceed via the ERAD pathway and through the proteasome. On the other hand, there is no significant decrease in the concentration of HBsAg even after prolonged cultivation [15] suggesting that the (lamellar) HBsAg deposits in the ER [14] are well protected from proteolytic degradation. Electron microscopic investigations revealed autophagic processes mainly related to peroxisome degradation and not to degradation of HBsAg deposits. Thus, the activation of autophagic processes also by ER-derived misfolded proteins as reported for mammalian [41] but also for fungal systems [46] might depend on specific protein properties and also on environmental conditions. During methanol-induced production of HBsAg in *P. pastoris*, autophagic processes are clearly related to peroxisome turnover most likely provoked by peroxisomal proteins and components damaged by reactive oxygen species.

## Methods

### Strain and growth conditions

The construction of the *P. pastoris* strain GS115 carrying 8-copies of the HBsAg structural gene under the control of the *aox1* promoter and displaying a Mut^S^ phenotype was described before [4]. Cells were grown on defined medium in a fed-batch procedure as described earlier [15]. High-level production of HBsAg was started after batch growth on glycerol through the addition of methanol to a final concentration of 6 g L^-1^. This methanol concentration was kept constant by continuous methanol feeding throughout the entire production phase [15].

### Sample preparation

Cells were harvested by centrifugation and washed with phosphate buffered saline to remove extracellular proteins and other contaminants. The remaining pellet was resuspended in extraction buffer (20 mmol L^-1^ Tris–HCl, pH 7.6, 10 mmol L^-1^ NaCl, 0.5 mmol L^-1^ deoxycholate, 1 μg mL^-1^ pepstatin). Cell disruption was accomplished by grinding in liquid nitrogen using a Mortar Grinder (RM 100, Retsch Gmbh &Co. KG, Germany). The cell debris was removed by centrifugation (22 000 × g, at 4°C for 30 min) and 1 mL of the subsequent supernatant treated for 15 min with 7 μL of nuclease mix (0.5 mg mL^-1^ DNase, 0.25 mg mL^-1^ RNase, 50 mmol L^-1^ MgCl_2_). Protein was precipitated with 30% TCA. Finally, the protein pellets were air-dried and dissolved in a solubilization buffer containing 7 mol L^-1^ urea, 2 mol L^-1^ thiourea, 4% (w/v) CHAPS, 1% (w/v) dithiothreitol (DTT), 20 mmol L^-1^ Tris, and 1% (v/v) Pharmalyte™ pH 3–10 (Amersham Biosciences). The total soluble protein concentration was determined using the BIO-RAD protein assay (BIO-RAD Lab., Hartfordshire, USA). The solubilized proteins were stored at −70°C until further analysis.

### Two-dimensional gel electrophoresis

Two-dimensional (2-D) gel electrophoresis was essentially carried out as described previously [17]. Briefly, the first-dimension using isoelectric focusing (IEF) was run with the IPGphor™ Isoelectric Focusing System (Amersham Biosciences) loading 300 μg protein sample onto Immobiline DryStrip gels of pH 3–10 (IPG strips, Amersham Biosciences) by in-gel rehydration. IEF was performed with the following setting: 30 V × 12 h, 300 V × 3 h, 600 V × 2 h, 1000 V × 1 h, gradient from 1000 V to 5000 V within 2 h, 5000 V × 2 h, gradient from 5000 V to 8000 V within 2 h, then 8000 V × 10 h. Prior to the second dimension (SDS-PAGE), the IPG strips were equilibrated and then transferred onto lab cast SDS-polyacrylamide gels (DALT multiple gel caster and DALT gradient maker, Amersham Biosciences). Proteins were separated on 12-16% linear gradient gels using the vertical separation unit Hoefer™ System (Amersham Biosciences). Subsequently, gels were stained using colloidal Coomassie Blue G-250 according to the “Blue silver” protocol [47]. The gels were then scanned (ScanMaker 9800 XL, Umax System GmbH, Germany) at 300 dpi resolution. Image analysis, namely protein spot detection, matching and quantification were performed using Proteomweaver™ 3.0 (Definens AG, Germany).

### In-gel trypsin digestion and peptide extraction

The intracellular proteome was analyzed at different cultivation time points by 2-D gel electrophoresis combined with Maldi-ToF analysis. A total of 136 protein spots were excised manually from Coomassie brilliant Blue stained 2-D gels, each spot was washed several times with 200 μl water, dehydrated in 50 μl acetonitrile, and dried in a vacuum concentrator (Eppendorf ® Vacufuge Concentrator 5301, Eppendorf AG, Hamburg). The gel pieces were treated with 100 mmol L^-1^ ammonium bicarbonate containing 20 mmol L^-1^ DTT at 56°C for 30 min and then with 100 mmol L^-1^ ammonium bicarbonate containing 55 mmol L^-1^ iodoacetamide in the dark at room temperature for 30 min. Acetonitrile was added in between the treatments to dehydrate the gel pieces. Finally, the gel pieces were washed twice with 100 mmol L^-1^ ammonium bicarbonate, dehydrated with acetonitrile and dried in the vacuum concentrator. In-gel digestion was carried out by incubation with 2 ng μL^-1^ trypsin (sequencing grade modified, Promega Corp.) in 50 mmol L^-1^ ammonium bicarbonate at 37°C overnight. Obtained peptides were extracted and then desalted with reversed-phased C-18 ZipTips (Millipore, Billerica, MA, USA) before application to the Maldi-ToF sample plates.

### Maldi-ToF MS analysis

Matrix-assisted laser desorption ionization time-of-flight mass spectrometry (Maldi-ToF MS) was employed to obtain the peptide mass fingerprint of a given protein. The concentrated peptide solution was mixed (1:1, v/v) with the MALDI loading solution (10 mg α-cyano-4-hydoxycinnamic acid, 400 μL acetonitrile and 600 μL 0.1% trifluoroacetic acid), loaded on the target and dried at room temperature. The molecular masses of the tryptic peptides were determined on a Bruker Ultraflex time-of-flight mass spectrometer (Bruker Daltonics GmbH, Germany).

### Data analysis

Peptide mass fingerprints obtained by the Maldi-ToF MS were processed using FlexAnalysis 2.0 (Bruker Daltonik GmbH, Germany) and used to search NCBInr database by using Mascot 2.10 software (http://www.matrixscience.com). The parameters used for the search were as follows: taxonomy: other Fungi, tryptic digestion, modifications were allowed for carbamidomethylation of cysteine (fixed modification) and methionine oxidation (variable modification), one missed cleavage site was allowed, all peptides monoisotopic, peptide tolerance at 100 ppm. Mascot scores (probability based MOWSE scores) and expect values were generated from the Mascot search program. All proteins with a Mowse score ≥ 70 were regarded as significant (p <0.05). Protein identification was based on the recently annotated *P. pastoris* GS115 genome sequence [48] and the gene name used in this study is according to *Pichia pastoris* strain GS115 (http://www.uniprot.org/). If no gene name was given for this strain the gene name is according to *Pichia pastoris* strain ATCC 76273 / CBS 7435 / CECT 11047 / NRRL Y-11430 / Wegner 21–1 or general *Pichia pastoris* (yeast) in case of 100% sequence identity (http://www.uniprot.org/). Image analysis from the scanned gels, namely protein spot detection, matching and quantification were performed using Proteomweaver™ 3.0 (Definiens AG, Germany). The spot volumes were computed and normalized for each spot on each gel in relation to the total spot volume of each 2D gel. To obtain comparable data, spot intensities were normalized using the log2 ratio of induced samples *versus* uninduced sample. Log2 fold changes above 0.6 (equivalent to a 1.5 fold changes) were considered significant.

### Ethanol analysis

Ethanol concentrations were determined by gas chromatography (Shimadzu 14B GC, Kyoto, Japan) using a column packed with Carbograph 1AW (20/120; 5% carbowax 20 M) (Alltech Associates Inc., Deerfield, IL, USA), column temperature 160°C, detector: flame ionization detector.

### Electron microscopy and image analysis

Electron microscopic studies were carried out essentially as described previously [14]. Mitochondrial and total cell area were determined from randomly chosen cells at the end of the glycerol batch phase (in total: 55 mitochondria in 12 cells) and after 112 hours of growth on methanol (in total: 67 mitochondria in 8 cells) using Image J Software (National Institutes of Health, Bethesda, Maryland, USA). For analysis of vacuolar morphology, 520 cells were randomly chosen for analysis (224 cells at the end of the glycerol batch phase, 160 cells 48 hours and 136 cells 112 hours after the onset of methanol feeding).

## Competing interests

The authors declare that they have no competing interests.

## Authors’ contributions

AV identified the proteins by Maldi-ToF, analyzed the data and prepared a first draft of the manuscript. HL prepared the electron micrographs. AA and CG carried out the cultivation. AA also prepared the 2D gels. MN contributed to protein identification by Maldi-ToF. NK was involved in the initial outline of the project. UR conceived and directed the study and prepared the final manuscript. All authors read and approved the final manuscript.

## Supplementary Material

Additional file 1 **All identified intracellular proteins.** The complete list of all identified proteins classified into functional categories and their log2 changes in response to methanol-induced high-level production of the HBsAg and a representative 2D gel image indicating the spots of all identified proteins are given. Click here for file

Additional file 2 **Identification of AOX1 in *P. pastoris *GS115 with a “Mut**^**s**^**phenotype”.** Unexpectedly AOX1 was identified in the supposedly Mut^s^ strain of *P. pastoris* GS115 producing high levels of HBsAg. Background information on AOX1 identification and discussion about Mut^S^ behavior of Mut^+^ strain is given. Click here for file
