# Supplementary material for: Physiological response of Pichia pastoris GS115 to methanol-induced high level production of the Hepatitis B surface antigen: catabolic adaptation, stress responses, and autophagic processes
Source: Microb Cell Fact. 2012 Aug 8;11:103. doi: 10.1186/1475-2859-11-103 (PMC3539919; doi:10.1186/1475-2859-11-103)
Supplement: Additional file 1 — All identified intracellular proteins. The complete list of all identified proteins classified into functional categories and their log2 changes in response to methanol-induced high-level production of the HBsAg and a representative 2D gel image indicating the spots of all identified proteins are given. [file 1475-2859-11-103-S1.pdf]

Additional file 1 All identified intracellular proteins. The complete list of all identified proteins classified into functional categories, a representative 2D gel image indicating the spots of all identified proteins and their log2 changes in response to methanol-induced high-level production of the HBsAg are given.

The complete list of all identified proteins classified into functional categories.

NCBI Accession-No [1]	Locus ID [1]	Gene name [2]	Protein name[2]	MM (kDa) [3]	p*I* [3]	Mascot Score	Spot No [4]
Functional category [1]							
**1. Metabolism**							
**1.1 Carbohydrate metabolism**							
**Methanol metabolism**							
XP_002494271	PAS_chr4_0821	*AOX1*	Alcohol oxidase	74.5	6.0	109	123*, 83*
XP_002492075	PAS_chr2-2_0131	*CTA1*	Catalase A	58.1	6.6	193	92*, 93*
XP_002493065	PAS_chr3_0832	*DAS1*	Transketolase (Dihydroxyacetone synthase), similar to Tkl2p	79.1	6.1	137	94*, 95*, 96*, 101*, 102*
XP_002493270	PAS_chr3_1028	*FLD1*	S-(hydroxymethyl) glutathione dehydrogenase	41.4	6.1	110	27*, 31*
XP_002493100	PAS_chr3_0867	*FGH1*	Non-essential intracellular esterase that can function as an S-formylglutathione hydrolase	33.4	6.3	222	5*
XP_002493171	PAS_chr3_0932	*FDH1*	NAD(+)-dependent formate dehydrogenase	40.4	6.6	191	2*, 3*, 4*, 29*, 30*, 85*, 103*
**Glycolysis**							
XP_002494063	PAS_chr4_0624	*EMI2*	Hexokinase	52.5	6.1	237	22*
XP_002491345	PAS_chr2-1_0437	*TDH1*	Glyceraldehyde-3-phosphate dehydrogenase, isozyme 3	35.7	6.2	154	114*, 117*
XP_002489713	PAS_chr1-1_0072	*FBA1*	Fructose 1,6-bisphosphate aldolase	39.9	6.0	108	33*
XP_002490411	PAS_chr1-4_0292	*PGK1*	3-Phosphoglycerate kinase	44.1	7.8	263	52*
XP_002493191	PAS_chr3_0951	*TPI1*	Triose phosphate isomerase	27.1	5.7	160	50*
XP_002493059	PAS_chr3_0826	*GMP1*	Tetrameric phosphoglycerate mutase	28.0	6.0	131	51*
XP_002492293	PAS_chr3_0082	*ENO2*	Enolase I	46.5	5.4	181	39*, 120*

	**Ethanol metabolism**						
CAY67035	PAS_c034_0018	*ADH*	Alcohol dehydrogenase	38.1	6.3	195	78*
XP_002491382	PAS_chr2-1_0472	*ADH3*	Mitochondrial alcohol dehydrogenase isozyme III	37.3	5.8	140	35*
	**Citrate cycle (TCA cycle)**						
XP_002489444	PAS_chr1-3_0104	*ACO1*	Aconitase	85.0	5.7	237	15*, 16*
XP_002491004	PAS_chr2-1_0120	*IDH2*	Subunit of mitochondrial NAD(+)-dependent isocitrate dehydrogenase	40.3	8.1	96	115*
XP_002492874	PAS_chr3_0647	*FUM1*	Fumarase	52.8	6.4	82	24*
XP_002491128	PAS_chr2-1_0238	*MDH1*	Mitochondrial malate dehydrogenase	36.5	5.3	230	34*, 46*
	**Pentose phosphate pathway**						
XP_002492495	PAS_chr3_0277	*6PGD [GND]*	6-Phosphogluconate dehydrogenase (decarboxylating)	54.2	5.9	209	21*
XP_002493617	PAS_chr4_0212	*RKI1*	Ribose-5-phosphate ketol-isomerase	23.6	6.0	226	69*
	**Sugar metabolism and others**						
XP_002490557	PAS_chr1-4_0426	*BGL2*	Endo-beta-1,3-glucanase	34.2	4.1	108	136*
	**Pyruvate metabolism**						
XP_002494290	PAS_chr4_0842	*GLO1*	Monomeric glyoxalase I	36.9	5.6	76	40*
XP_002491701	PAS_chr2-1_0767	*acsB [ACS1]*	Acetyl-coA synthetase isoform	74.1	5.7	88	18*, 19*
**1.2 Energy Metabolism**							
	**Oxidative phosphorylation**						
XP_002489364	PAS_chr1-3_0028	*IPP1*	Cytoplasmic inorganic pyrophosphatase (PPase)	32.3	5.3	214	65*, 134*
XP_002493409	PAS_c131_0021	*[VMA2]*	Vacuolar ATP synthase subunit B	55.4	5.3	169	14*
XP_002494179	PAS_chr4_0737	*MCR1*	Mitochondrial NADH-cytochrome b5 reductase	33.6	8.8	183	54*, 105*
**1.3 Nucleotide metabolism**							
XP_002492473	PAS_chr3_0257	*ADK1*	Adenylate kinase	28.2	7.0	122	106*
XP_002492154	PAS_chr2-2_0059	*YNK1*	Nucleoside diphosphate kinase	17.0	6.2	111	89*

**1.4 Amino acid metabolism**							
XP_002489848	PAS_chr1-1_0200	*AAT1*	Cytosolic aspartate aminotransferase	47.9	6.7	200	28*, 25*
XP_002492036	PAS_chr2-2_0168	*[LYS12]*	Homo-isocitrate dehydrogenase	40.1	6.0	121	41*
XP_002490090	PAS_chr1-1_0432	*ILV5*	Acetohydroxyacid reductoisomerase	44.4	7.7	94	43*
XP_002491046	PAS_chr2-1_0160	*metE [MET6]*	Cobalamin-independent methionine synthase	85.9	5.9	228	17*, 118*
XP_002492638	PAS_chr3_0410	*CAR2*	L-Ornithine transaminase	47.5	5.9	100	26*
XP_002493966	PAS_chr4_0974	*[AAT2]*	Aspartate aminotransferase	42.5	7.2	174	133*
XP_002492166	PAS_chr2-2_0048	*[LPD1]*	Dihydrolipoamide dehydrogenase	52.6	6.3	110	119*
**1.5 Metabolism of cofactors and vitamins**							
XP_002493470	PAS_chr4_0065	*THI5*	Protein involved in synthesis of the thiamine precursor hydroxymethylpyrimidine (HMP)	38.7	6.3	140	116*
XP_002493989	PAS_chr4_0550	-	Pyridoxine 4-dehydrogenase	36.3	5.6	192	42*
XP_002492582	PAS_chr3_0361	*[PNC1]*	Nicotinamidase	24.7	5.3	126	70*
**2. Genetic information processing**							
**2.1 Transcription and translation**							
XP_002489515	PAS_chr1-3_0172	*EGD1*	Subunit beta1 of the nascent polypeptide-associated complex (NAC)	17.9	4.8	76	72*
XP_002489404	PAS_chr1-3_0068	*RPP0*	Conserved ribosomal protein P0 similar to rat P0, human P0, and E. coli L10e	33.7	4.6	110	63*
XP_002493616	PAS_chr4_0211	*[RPS7B]*	Protein component of the small (40S) ribosomal subunit. nearly identical to Rps7Bp	21.3	9.2	90	112*
XP_002491009	PAS_chr2-1_0812	*EFT1*	Elongation factor 2	93.9	6.3	83	100*
XP_002492789	PAS_chr3_0562	*TUF1*	Mitochondrial translation elongation factor Tu	47.0	5.6	103	44*
XP_002493313	PAS_chr3_1071	*CAM1*	Translation elongation factor EF-1 gamma	24.3	6.4	111	107*, 132*
**2.2 Sorting and degradation**							
XP_002492588	PAS_chr3_0365	*SSC1*	Mitochondrial matrix ATPase	69.7	5.4	98	99*
XP_002490239	PAS_chr1-4_0130	-	Heat shock protein Hsp90	80.9	4.8	100	122*
XP_002490142	PAS_chr1-4_0027	*COF1*	Cofilin	18.2	6.1	97	71*
XP_002493333	PAS_chr3_1087	*APR1 [PEP4]*	Vacuolar aspartyl protease (proteinase A)	44.5	4.6	134	86*

XP_002490155	PAS_chr1-4_0043	*SBA1*	Co-chaperone that binds to and regulates Hsp90 family chaperones	22.5	4.3	77	113*
XP_002489443	PAS_chr1-3_0102	*clpB [HSP104]*	Heat shock protein that cooperates with Ydj1p (Hsp40) and Ssa1p (Hsp70)	100.6	5.3	160	84*
XP_002494360.1	PAS_FragD_0026	*[CDC48]*	ATPase in ER, nuclear membrane and cytosol with homology to mammalian p97	91.4	4.8	216	1*
XP_002492868	PAS_chr3_0640	*TFS1*	Carboxypeptidase Y inhibitor	29.8	7.8	155	6*, 7*
**2.3 Folding catalysts**							
CAC33587	PAS_chr4_0844	*PDI*	Protein disulphide isomerase	58.1	4.6	160	11*
XP_002493562	PAS_chr4_0158	*CPNA [HSP60]*	Tetradecameric mitochondrial chaperonin	60.5	5.1	180	12*
XP_002489608	PAS_chr1-3_0264	*[CPR1]*	Cytoplasmic peptidyl-prolyl cis-trans isomerase (cyclophilin)	18.1	6.1	103	56*
XP_002490274	PAS_chr1-4_0163	*[HSP10]*	10 kDa chaperonin	11.1	9.0	82	82*
**3. Cellular processes**							
**3.1 Transport and catabolism**							
XP_002490181	PAS_chr1-4_0071	*SOD2*	Mitochondrial superoxide dismutase	25.2	7.9	111	76*
**3.2 Cell growth and death**							
XP_002493242	PAS_chr3_1001	*TUP1*	General repressor of transcription, forms complex with Cyc8p	66.3	6.0	135	20*
XP_002490416	PAS_chr1-4_0297	*[STM1]*	Suppressor protein STM1	29.7	9.7	102	87*
**4. Others**							
**4.1 Stress response**							
XP_002493863	PAS_chr4_0433	-	NADPH-dependent alpha-keto amide reductase	33.7	5.6	113	73*
XP_002490683	PAS_chr1-4_0547	*PMP20*	Peroxiredoxin	18.4	9.5	178	9*
XP_002493699	PAS_chr4_0284	*TRX2*	Cytoplasmic thioredoxin isoenzyme of the thioredoxin system	11.4	4.9	110	80*
XP_002492079	PAS_chr2-2_0127	*CCP1*	Mitochondrial cytochrome-c peroxidase	42.0	6.6	197	66*
XP_002491803	PAS_chr2-2_0382	*GPX1 [HYR1]*	Thiol peroxidase (Glutathione peroxidase)	18.4	6.2	164	8*
XP_002493423	PAS_chr4_0018	*CYC1*	Cytochrome c, isoform 1	12.2	9.6	94	81*
BAH80186	PAS_chr2-2_0480	*TRR1*	Thioredoxin reductase 1	35.3	5.5	141	67*

5. Unclassified

XP_002491489	PAS_chr2-1_0573	-	Putative xylose and arabinose reductase	32.2	6.1	167	75*
XP_002491564	PAS_chr2-1_0640	-	Putative protein of unknown function	21.7	6.3	119	129*
XP_002491418	PAS_chr2-1_0853	-	Hypothetical protein	57.0	6.0	119	121*
XP_002489760	PAS_chr1-1_0118	-	Hypothetical protein	46.3	7.0	77	127*
XP_002490519	PAS_chr1-4_0675	-	Hypothetical protein	53.8	6.7	96	23*
XP_002491208	PAS_chr2-1_0313	-	Bifunctional enzyme with alcohol dehydrogenase and glutathione-dependent formaldehyde dehydrogenase	39.1	5.8	177	37*
XP_002491071	PAS_chr2-1_0183	-	Acyl-protein thioesterase responsible for depalmitoylation of Gpa1p	24.3	6.3	102	130*
XP_002491989	PAS_chr2-2_0208	-	Hypothetical protein	18.6	6.5	85	10*
XP_002491901	PAS_chr2-2_0289	-	Hypothetical protein	28.3	4.8	125	64*
XP_002492584	PAS_chr3_0362	-	Mitochondrial peculiar membrane protein 1	32.7	5.7	92	68*
XP_002493742	PAS_chr4_0320	*MYH4*	Myosin-4	92.6	4.9	152	59*

1 Accession numbers and locus IDs are according to the NCBI Reference Sequence database (http://www.ncbi.nlm.nih.gov/RefSeq/). Functional classifications are mostly according to KEGG PATHWAY database (http://www.genome.jp/kegg/metabolism.html).

2 Gene/protein names are according to the sequenced genome of *P. pastoris* GS115 (http://www.uniprot.org/). If no name was found for *P. pastoris* GS115, a Blast search of the respective *P. pastoris* GS115 gene/protein against different *P. pastoris* strains was carried out in the following order: search against ATCC 76273 / CBS 7435 / CECT 11047 / NRRL Y-11430 / Wegner 21-1 and *P. pastoris* (yeast). Gene/protein names from other *P. pastoris* strains were only adopted in case of 100% sequence identity (http://www.uniprot.org/). Gene names in brackets are from *S. cerevisiae* if the names differ for both yeasts (http://www.uniprot.org/).

3 Theoretical molecular mass (MM) and isoelectric point (p*I*) not considering potential posttranslational modifications.

4 "Spot-No" indicates the serial number of all identified protein spots of the intracellular proteome of *P. pastoris* GS115. Proteins appearing in multiple spots indicate the existence of isoforms which probably derive from posttranslational modification events such as phosphorylation, glycosylation or limited proteolysis. A representative 2D gel of the intracellular proteome from a cell sample taken after 114 hours of growth on methanol with the respective spot numbers is shown below. Spot numbers in this table and the following gel are marked by an asterix (*) for simplified searchability.

A representative 2D gel of the intracellular proteome of *P. pastoris* GS115 producing HBsAg.

The sample was taken after 114 h of growth on methanol. Spot numbers correspond to numbers in the table above and are marked by an asterix (*) for simplified searchability.

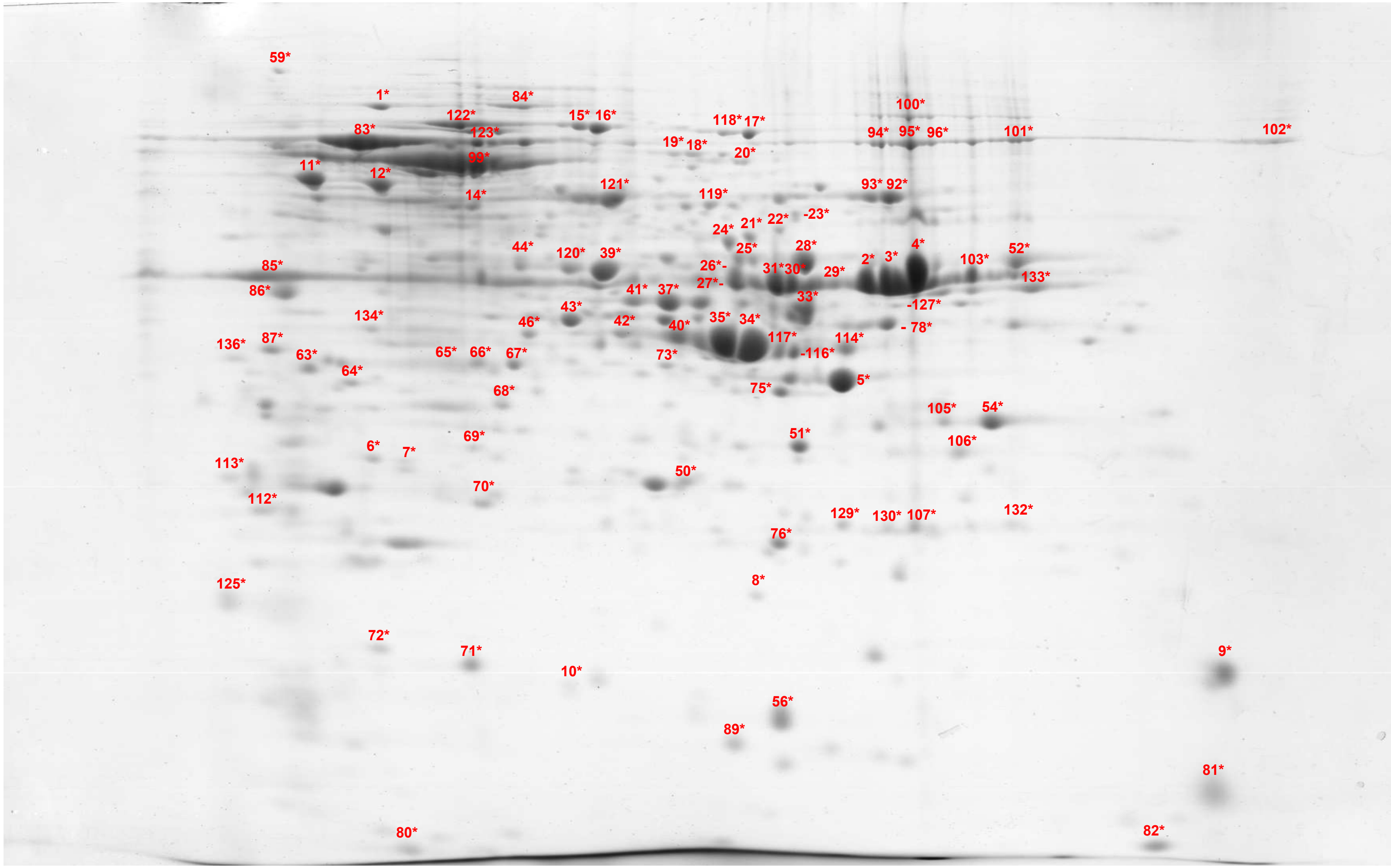

Change of the intracellular proteome in response to high-level methanol-induced production of HBsAg. Samples before methanol addition at the end of the glycerol batch phase and 4, 16, 72 and 114 hours after the onset of methanol feeding were analyzed and the protein changes in response to methanol-induced HBsAg production are shown (numbers. log2 changes; n.a. not analyzable, color code: light orange to red, increase, light green to dark green: decrease, yellow: no significant change).

Functional category	Gene name	Gene ID	0h [1]	4h	16h	72h	114h
**Carbohydrate metabolism**							
Methanol metabolism	*AOX1*	PAS_chr4_0821	0.0	10.8	11.6	10.8	12.0
	CTA1	PAS_chr2-2_0131	0.0	0.6	1.0	1.0	1.5
	DAS1	PAS_chr3_0832	0.0	0.5	3.8	3.2	3.1
	FLD1	PAS_chr3_1028	0.0	9.1	10.0	9.0	10.7
	FGH1	PAS_chr3_0867	0.0	2.0	3.8	4.5	4.2
	FDH1	PAS_chr3_0932	0.0	10.6	12.7	13.4	13.5
Glycolysis	*EMI2*	PAS_chr4_0624	0.0	-0.7	-1.8	-3.8	-1.8
	TDH1	PAS_chr2-1_0437	0.0	-0.2	0.4	-0.7	0.0
	FBA1	PAS_chr1-1_0072	0.0	0.1	-0.4	-0.5	-0.8
	PGK1	PAS_chr1-4_0292	0.0	0.1	-0.9	-0.7	-0.7
	TPI1	PAS_chr3_0951	0.0	-0.1	-1.3	-2.0	-1.7
	GMP1	PAS_chr3_0826	0.0	0.0	-0.2	0.1	-0.8
	ENO2	PAS_chr3_0082	0.0	-0.4	-0.5	-0.2	-0.7
Ethanol metabolism	*ADH*	PAS_c034_0018	0.0	0.8	0.2	0.8	0.7
	ADH3	PAS_chr2-1_0472	0.0	0.5	0.7	1.0	0.6
Citrate cycle (TCA)	*ACO1*	PAS_chr1-3_0104	0.0	-0.3	-0.6	0.6	0.3
	IDH2	PAS_chr2-1_0120	0.0	0.3	-0.8	-0.4	-0.6
	FUM1	PAS_chr3_0647	0.0	-0.1	-0.7	-0.1	-0.7
	MDH1	PAS_chr2-1_0238	0.0	0.8	1.1	1.7	1.0
Pentose phosphate pathway	*6PGD [GND]*	PAS_chr3_0277	0.0	0.0	n.a	n.a	-1.0
	RKI1	PAS_chr4_0212	0.0	-0.2	1.0	1.2	0.2
Sugar metabolism	*BGL2*	PAS_chr1-4_0426	0.0	-1.2	-1.4	-0.8	-1.3
Pyruvate metabolism	*GLO1*	PAS_chr4_0842	0.0	0.5	0.3	1.3	1.2
	acsB [ACS1]	PAS_chr2-1_0767	0.0	0.0	-1.8	-1.6	-0.9

Functional category	Gene name	Gene ID	0h	4h	16h	72h	114h
**Energy metabolism**							
Oxidative phosphorylation	*IPP1*	PAS_chr1-3_0028	0.0	1.1	2.9	0.4	0.0
	[VMA2]	PAS_c131_0021	0.0	0.0	0.0	0.4	-0.4
	MCR1	PAS_chr4_0737	0.0	0.1	-0.4	0.9	0.4
**Nucleotide metabolism**							
	ADK1	PAS_chr3_0257	0.0	0.0	-1.1	0.0	-0.2
	YNK1	PAS_chr2-2_0059	0.0	-0.1	-1.2	-0.3	-1.2
**Amino acid metabolism**							
	AAT1	PAS_chr1-1_0200	0.0	0.3	0.9	1.3	1.1
	[LYS12]	PAS_chr2-2_0168	0.0	-0.3	-0.6	1.0	-0.4
	ILV5	PAS_chr1-1_0432	0.0	0.0	-0.4	0.1	-0.5
	metE [MET6]	PAS_chr2-1_0160	0.0	0.2	-4.2	-5.2	-1.8
	CAR2	PAS_chr3_0410	0.0	0.0	0.2	-0.1	n.a
	[AAT2]	PAS_chr4_0974	0.0	1.0	0.6	0.9	1.2
	[LPD1]	PAS_chr2-2_0048	0.0	0.5	-1.1	-0.1	-0.5
**Metabolism of cofactors and vitamins**							
	THI5	PAS_chr4_0065	0.0	1.1	1.0	-1.0	-0.3
	-	PAS_chr4_0550	0.0	-0.2	-0.2	-0.1	-0.7
	[PNC1]	PAS_chr3_0361	0.0	-1.3	-1.2	-0.2	-0.7
**Genetic information processing**							
Transcription and translation	*EGD1*	PAS_chr1-3_0172	0.0	0.5	0.4	0.5	-0.5
	RPP0	PAS_chr1-3_0068	0.0	7.9	6.9	7.6	7.0
	[RPS7B]	PAS_chr4_0211	0.0	-0.3	-0.3	-0.5	n.a
	EFT1	PAS_chr2-1_0812	0.0	n.a	-3.4	n.a	-0.8
	TUF1	PAS_chr3_0562	0.0	0.0	-0.4	-0.1	-1.9
	CAM1	PAS_chr3_1071	0.0	-1.4	-1.3	0.7	0.8
Sorting and degradation	*SSC1*	PAS_chr3_0365	0.0	0.3	1.0	1.0	-0.3
	-	PAS_chr1-4_0130	0.0	0.7	0.4	1.0	1.2
	COF1	PAS_chr1-4_0027	0.0	0.0	-0.3	0.3	-0.5
	APR1 [PEP4]	PAS_chr3_1087	0.0	-0.1	2.5	3.4	3.2
	SBA1	PAS_chr1-4_0043	0.0	-0.8	-0.3	-0.1	-1.0
	clpB [HSP104]	PAS_chr1-3_0102	0.0	8.2	5.7	n.a	7.1
	[CDC48]	PAS_FragD_0026	0.0	1.4	1.3	1.0	0.8
	TFS1	PAS_chr3_0640	0.0	0.1	-0.6	-0.1	-1.3

Functional category	Gene name	Gene ID	0h	4h	16h	72h	114h
Folding catalysts	*PDI*	PAS_chr4_0844	0.0	0.7	1.3	1.8	1.7
	CPNA [HSP60]	PAS_chr4_0158	0.0	0.1	0.4	0.8	0.1
	[CPR1]	PAS_chr1-3_0264	0.0	1.3	1.2	1.3	0.3
	[HSP10]	PAS_chr1-4_0163	0.0	0.1	0.5	0.7	0.8
**Cellular processes**							
Transport and catabolism	*SOD2*	PAS_chr1-4_0071	0.0	0.5	-0.1	0.2	0.2
Cell growth and death	*TUP1*	PAS_chr3_1001	0.0	0.3	-1.2	-1.5	-1.9
	[STM1]	PAS_chr1-4_0297	0.0	0.3	-0.3	0.4	-0.6
**Others**							
Stress response	-	PAS_chr4_0433	0.0	-0.5	-1.5	-2.0	-1.9
	PMP20	PAS_chr1-4_0547	0.0	8.9	10.1	10.0	9.7
	TRX2	PAS_chr4_0284	0.0	0.3	0.0	0.3	-0.9
	CCP1	PAS_chr2-2_0127	0.0	0.7	0.1	0.4	-1.1
	GPX1 [HYR1]	PAS_chr2-2_0382	0.0	0.6	0.3	0.6	-0.5
	CYC1	PAS_chr4_0018	0.0	0.3	-0.1	0.6	0.8
	TRR1	PAS_chr2-2_0480	0.0	0.2	-0.3	-0.4	-1.3
Unclassified	-	PAS_chr2-1_0573	0.0	0.0	0.4	1.1	0.7
	-	PAS_chr2-1_0640	0.0	-0.1	-0.7	-0.5	-1.8
	-	PAS_chr2-1_0853	0.0	1.0	1.0	-0.2	2.6
	-	PAS_chr1-1_0118	0.0	-1.6	-1.0	-0.2	-1.3
	-	PAS_chr1-4_0675	0.0	-0.4	-1.7	-2.5	-1.1
	-	PAS_chr2-1_0313	0.0	-0.1	-0.5	-0.4	0.3
	-	PAS_chr2-1_0183	0.0	0.4	1.8	1.9	2.0
	-	PAS_chr2-2_0208	0.0	1.8	2.5	1.0	1.1
	-	PAS_chr2-2_0289	0.0	-0.2	-1.6	0.2	-1.2
	-	PAS_chr3_0362	0.0	-1.1	-0.8	0.6	-0.1
	MYH4	PAS_chr4_0320	0.0	-0.3	-2.0	-0.1	-1.5

[1] When no protein spot was detectable at the end of the glycerol batch phase (methanol metabolism), a virtual number of 0.001 was taken for the spot intensity to allow the calculation of log2 values (spot intensities were normalized using the log2 ratio of induced samples *versus* uninduced sample).
