# Supplementary material for: Physiological response of Pichia pastoris GS115 to methanol-induced high level production of the Hepatitis B surface antigen: catabolic adaptation, stress responses, and autophagic processes
Source: Microb Cell Fact. 2012 Aug 8;11:103. doi: 10.1186/1475-2859-11-103 (PMC3539919; doi:10.1186/1475-2859-11-103)
Supplement: Additional file 2 — Identification of AOX1 in P. pastoris GS115 with a “Mutsphenotype”. Unexpectedly AOX1 was identified in the supposedly Muts strain of P. pastoris GS115 producing high levels of HBsAg. Background information on AOX1 identification and discussion about MutS behavior of Mut+ strain is given. [file 1475-2859-11-103-S2.pdf]

Additional file 2 Identification of AOX1 in *P. pastoris* GS115 with a "Mut^s phenotype"

Results and Discussion

The HBsAg producing strain was originally identified as a Mut^s (**m**ethanol **u**tilization **s**low) strain devoid of the *aox1* gene [1]. Thus, the identification of AOX1 in the intracellular proteome of *P. pastoris* GS115 after methanol-induced production of HBsAg by Maldi-ToF MS was unexpected (Figure 1). To confirm the presence of AOX1 and exclude a misidentification of AOX2, peptides which sequence differences in AOX1 and AOX2 were selected for sequencing by MS/MS. These results confirmed the correct identification of AOX1 (Figure 1). Finally, the presence of the entire *aox1* gene in the genome was confirmed by PCR analysis (Figure 2). The first attempt to verify the presence of the entire *aox1* gene by PCR with external primers using the Taq DNA polymerase failed; only the multicopy *HBsAg* gene was amplified under the applied conditions (Figure 2A, lane 1). However, the presence of *aox1* or at least parts of the *aox1* gene was confirmed by using internal primers (Figure 2A, lane 2). Finally, the presence of the entire *aox1* gene in the genome was confirmed by PCR analysis using a high performance DNA polymerase and a modified PCR protocol (see Materials and Methods for details).

Our results clearly show that a Mut^s phenotype, namely slow growth on methanol, does not necessarily prove the absence of a functional *aox1* gene. For example, slow growth on methanol was also observed with of an $aox1^+$ strain carrying multiple copies of a gene encoding a porcine insulin precursor [2]. Thus, high level expression of foreign genes can result in a metabolic burden and cellular stress response which may lead to impaired growth on methanol despite the presence of functional *aox1*.

Moreover, the absence of a visible *aox1* PCR product using external *aox1* primers does not necessarily proof the absence of *aox1*. It is advised to use internal *aox1* primers to allow selective *aox1* amplification this way preventing interference through selective amplification of multiple copies of shorter foreign DNA fragments. Alternatively, PCR protocols can be optimized to allow simultaneous amplification of *aox1* and the foreign DNA fragment.

Material and Methods

Maldi-ToF MS and MS/MS analysis

Maldi-ToF MS and MS/MS analysis and sample preparation were carried out as described in the main manuscript. AOX1 was identified from several gels with samples from different cultivations (shake flask and bioreactor) using the same transformant [1,3,4]. Three peptides, which reveal sequence differences in AOX1 and AOX2, were sequenced by MS/MS (Figure 1). The theoretical mass of AOX1 and AOX2 and corresponding peptides were predicted using [http://web.expasy.org/peptide_mass/]. The amino acid sequence of AOX1 and AOX2 are given in Figure 3.

PCR analysis and DNA sequencing

Cells were lysed in TRIzol reagent (Invitrogen, Carlsbad, CA) and the DNA isolated for PCR analysis according to the manufacturer´s instruction (www.invitrogen.com). The following primers were employed for PCR: AOX1 external primer, AOX1 internal primer and HBsAg internal primer (sequence given below). Two PCR protocols were used: the first PCR was carried out using the Taq DNA polymerase (Takara^{TM}, Japan) and the following conditions: initial denaturation at 95°C for 30 sec; annealing at 55°C for 30 sec and extension at 72°C for 30 sec with 35 cycles of amplification. The second PCR was carried out using a high performance polymerase (Ex Taq DNA polymerase, Takara^{TM}, Japan) and the following conditions: initial denaturation at 95°C for 30 sec; annealing at 57°C for 30 sec and extension at 72°C for 1 min with 35 circles of amplification. The second protocol allowed the simultaneous amplification of the shorter *HBsAg* and the longer *aox1* sequence. The PCR products were separated by agarose electrophoresis, the DNA extracted and subjected to sequence analysis. The identity of *aox1* was confirmed by sequencing (Eurofins MWG GmbH) (data not shown).

DNA sequence of *aox1* from *P. pastoris* strain GS115 with 5’ promoter and 3’ terminator

AGATCTAACATCCAAAGACGAAAGGTTGAATGAAACCTTTTTGCCATCCGACATCCACAGGTCCATTCTCACAC
ATAAGTGCCAAACGCAACAGGAGGGGATACACTAGCAGCAGACCGTTGCAAACGCAGGACCTCCACTCCTCTT
CTCCTCAACACCCACTTTTGCCATCGAAAAACCAGCCCAGTTATTGGGCTTGATTGGAGCTCGCTCATTCCAATT
CCTTCTATTAGGCTACTAACACCATGACTTTATTAGCCTGTCTATCCTGGCCCCCCTGGCGAGGTTCATGTTTGT
TTATTTCCGAATGCAACAAGCTCCGCATTACACCCGAACATCACTCCAGATGAGGGCTTTCTGAGTGTGGGGTC
AAATAGTTTCATGTTCCCCAAATGGCCCAAAACTGACAGTTTAAACGCTGTCTTGGAACCTAATATGACAAAAG
CGTGATCTCATCCAAGATGAACTAAGTTTGGTTCGTTGAAATGCTAACGGCCAGTTGGTCAAAAAGAAACTTCC
AAAAGTCGGCATACCGTTTGTCTTGTTTGGTATTGATTGACGAATGCTCAAAAATAATCTCATTAATGCTTAGC
GCAGTCTCTCTATCGCTTCTGAACCCCGGTGCACCTGTGCCGAAACGCAAATGGGGAAACACCCGCTTTTTGGA
TGATTATGCATTGTCTCCACATTGTATGCTTCCAAGATTCTGGTGGGAATACTGCTGATAGCCTAACGTTCATGA
TCAAAATTTAACTGTTCTAACCCCTACTTGACAGCAATATATAAACAGAAGGAAGCTGCCCTGTCTTAAACCTTT
TTTTTTATCATCATTATTAGCTTACTTTCATAATTGCGACTGGTTCCAATTGACAAGCTTTTGATTTTAACGACTTT
TAACGACAACTTGAGAAGATCAAAAAACAACTAATTATTCGAAACGATGGCTATCCCCGAAGAGTTTGATATCC
TAGTTCTAGGTGGTGGATCCAGTGGATCCTGTATTGCCGGAAGATTGGCAAACTTGGACCACTCCTTGAAAGT
TGGTCTTATCGAAGCAGGTGAGAACAACCTCAACAACCCATGGGTCTACCTTCCAGGTATTTACCCAAGAAACA
TGAAGTTGGACTCCAAGACTGCTTCCTTCTACACTTCTAACCCATCTCCTCACTTGAATGGTAGAAGAGCCATTG
TTCCATGTGCTAACGTCTTGGGTGGTGGTTCTTCTATCAACTTCATGATGTACACCAGAGGTTCTGCTTCTGATT
ACGATGACTTCCAAGCCGAGGGCTGGAAAACCAAGGACTTGCTTCCATTGATGAAAAAGACTGAGACCTACCA
AAGAGCTTGCAACAACCCTGACATTCACGGTTTCGAAGGTCCAATCAAGGTTTCTTTCGGTAACTACACCTACC
CAGTTTGCCAGGACTTCTTGAGGGCTTCTGAGTCCCAAGGTATTCCATACGTTGACGACTTGGAAGACTTGGTT
ACTGCTCACGGTGCTGAACACTGGTTGAAGTGGATCAACAGAGACACTGGTCGTCGTTCCGACTCTGCTCATG
CATTTGTCCACTCTACTATGAGAAACCACGACAACTTGTACTTGATCTGTAACACGAAGGTCGACAAAATTATT
GTCGAAGACGGAAGAGCTGCTGCTGTTAGAACCGTTCCAAGCAAGCCTTTGAACCCAAAGAAGCCAAGTCACA
AGATCTACCGTGCTAGAAAGCAAATCGTTTTGTCTTGTGGTACCATCTCCTCTCCATTGGTTTTGCAAAGATCCG
GTTTTGGTGACCCAATCAAGTTGAGAGCCGCTGGTGTTAAGCCTTTGGTCAACTTGCCAGGTGTCGGAAGAAA
CTTCCAAGACCACTACTGTTTCTTCAGTCCTTACAGAATCAAGCCTCAGTACGAGTCTTTCGATGACTTCGTCCG
TGGTGATGCTGAGATTCAAAAGAGAGTCTTTGACCAATGGTACGCCAATGGTACTGGTCCTCTTGCCACTAAC
GGTATCGAAGCTGGTGTCAAGATCAGACCAACACCAGAAGAACTCTCTCAAATGGACGAATCCTTCCAGGAGG
GTTACAGAGAATACTTCGAAGACAAGCCAGACAAGCCAGTTATGCACTACTCCATCATTGCTGGTTTCTTCGGT
GACCACACCAAGATTCCTCCTGGAAAGTACATGACTATGTTCCACTTCTTGGAATACCCATTCTCCAGAGGTTCC
ATTCACATTACCTCCCCAGACCCATACGCAGCTCCAGACTTCGACCCAGGTTTCATGAACGATGAAAGAGACAT
GGCTCCTATGGTTTGGGCTTACAAGAAGTCTAGAGAAACCGCTAGAAGAATGGACCACTTTGCCGGTGAGGTC
ACTTCTCACCACCCTCTGTTCCCATACTCATCCGAGGCCAGAGCCTTGGAAATGGATTTGGAGACCTCTAATGC
CTACGGTGGACCTTTGAACTTGTCTGCTGGTCTTGCTCACGGTTCTTGGACTCAACCTTTGAAGAAGCCAACTG
CAAAGAACGAAGGCCACGTTACTTCGAACCAGGTCGAGCTTCATCCAGACATCGAGTACGATGAGGAGGATG
ACAAGGCCATTGAGAACTACATTCGTGAGCACACTGAGACCACATGGCACTGTCTGGGAACCTGTTCCATCGG
TCCAAGAGAAGGTTCCAAGATCGTCAAATGGGGTGGTGTTTTGGACCACAGATCCAACGTTTACGGAGTCAAG
GGCTTGAAGGTTGGTGACTTGTCCGTGTGCCCAGACAATGTTGGTTGTAACACCTACACCACCGCTCTTTTGAT
CGGTGAAAAGACTGCCACTTTGGTTGGAGAAGATTTAGGATACTCTGGTGAGGCCTTAGACATGACTGTTCCT
CAGTTCAAGTTGGGCACTTACGAGAAGACCGGTCTTGCTAGATTCTAATCAAGAGGATGTCAGAATGCCATTT
GCCTGAGAGATGCAGGCTTCATTTTTGATTACTTTTTTATTTGTAACCTATATAGTATAGGATTTTTTTTGTCATT
TTGTTTCTTCTCGTACGAGCTTGCTCCTGATCAGCCTATCTCGCAGCTGATGAATATCTTGTGGTAGGGGTTTGG
GAAAATCATTCGAGTTTGATGTTTTTCTTGGTATTTCCCACTCCTCTTCAGAGTACAGAAGATTAAGTGAGACGT
TCGTTTGTGC

Color code:

Red underlined – 5’ promoter, 940 bp (1-940 bp)

Black – *aox1* gene, 1992bp (941- 2932 bp)

Purple underlined – 3’ terminator, 338 bp (2865 – 3193 bp)

Green background – External primer binding sites, 2105 bp between primers (855-875, 2939-2959)

Primers used for PCR

f *aox1* external – 5´ GACTGGTTCCAATTGACAAGC 3´

r *aox1* external – 5´ GCAAATGGCATTCTGACATCC 3´

Blue background – Internal primer binding sites, 507bp between primers (996-1015, 1483-1502)

Primers used for PCR

f aox 1 internal – 5´ CCTGTATTGCCGGAAGATTG 3´

r *aox*1 internal – 5´ CAGCACCGTGAGCAGTAACC 3´

Yellow background – sequence difference of *aox1* and *aox2* within the two internal primers

***HBsAg* sequence**

ATG GAG AAC ATC ACA TCA GGA TTC CTA GGA CCC CTG CTC GTG TTA CAG GCG GGG TTT TTC TTG TTG ACA AGA ATC CTC ACA ATA CCG CAG AGT CTA GAC TCG TGG TGG GCT TCT CTC AAT TTT CTA GGG GGA TCA CCC GTG TGT CTT GGC CAA AAT TCG CAG TCC CCA ACC TCC AAT CAC TCA CCA ACC TCC TGT CCT CCA ATT TGT CCT GGT TAT CGC TGG ATG TGT CTG CGG CGT TTT ATC ATA TTC CTC TTC ATC CTG CTG CTA TGC CTC ATC TTC TTA TTG GTT CTT CTG GAT TAT CAA GGT ATG TTG CCC GTT TGT TCT CTA ATT CCA GGA TCA ACA ACA ACC AGT ACG GGA CCA TGC AAA ACC TGC ACG ACT CCT GCT CAA GGC AAC TCT ATG TTT CCC TCA TGT TGC TGT ACA AAA CCT ACG GAT GGA AAT TGC ACC TGT ATT CCC ATC CCA TCG TCC TGG GCT TTC GCA AAA TAC CTA TGG GAG TGG GCC TCA GTC CGT TTC TCT TGG CTC AGT TTA CTA GTG CCA TTT GTT CAG TGG TTC GTA GGG CTT TCC CCC ACT GTT TGG CTT TCA GCT ATA TGG ATG ATG TGG TAT TGG GGG CCA AGT CTG TAC AGC ATC GTG AGT CCC TTT ATA CCG CTG TTA CCA ATT TTC TTT TGT CTC TGG GTA TAC ATT TAA

Color code:

Blue underlined – start codon

Black – *HBsA*g gene

Red underlined – stop codon

Green background – *HBsAg* internal primer binding sites, 447bp between primers (162-183, 588-608)

Primers used for PCR

f *HBsAg* internal – 5´ GTCCCCAACCTCCAATCACTCA 3´

r *HBsAg* internal – 5´ GGCCCCCAATACCACATCATC 3´

Figures

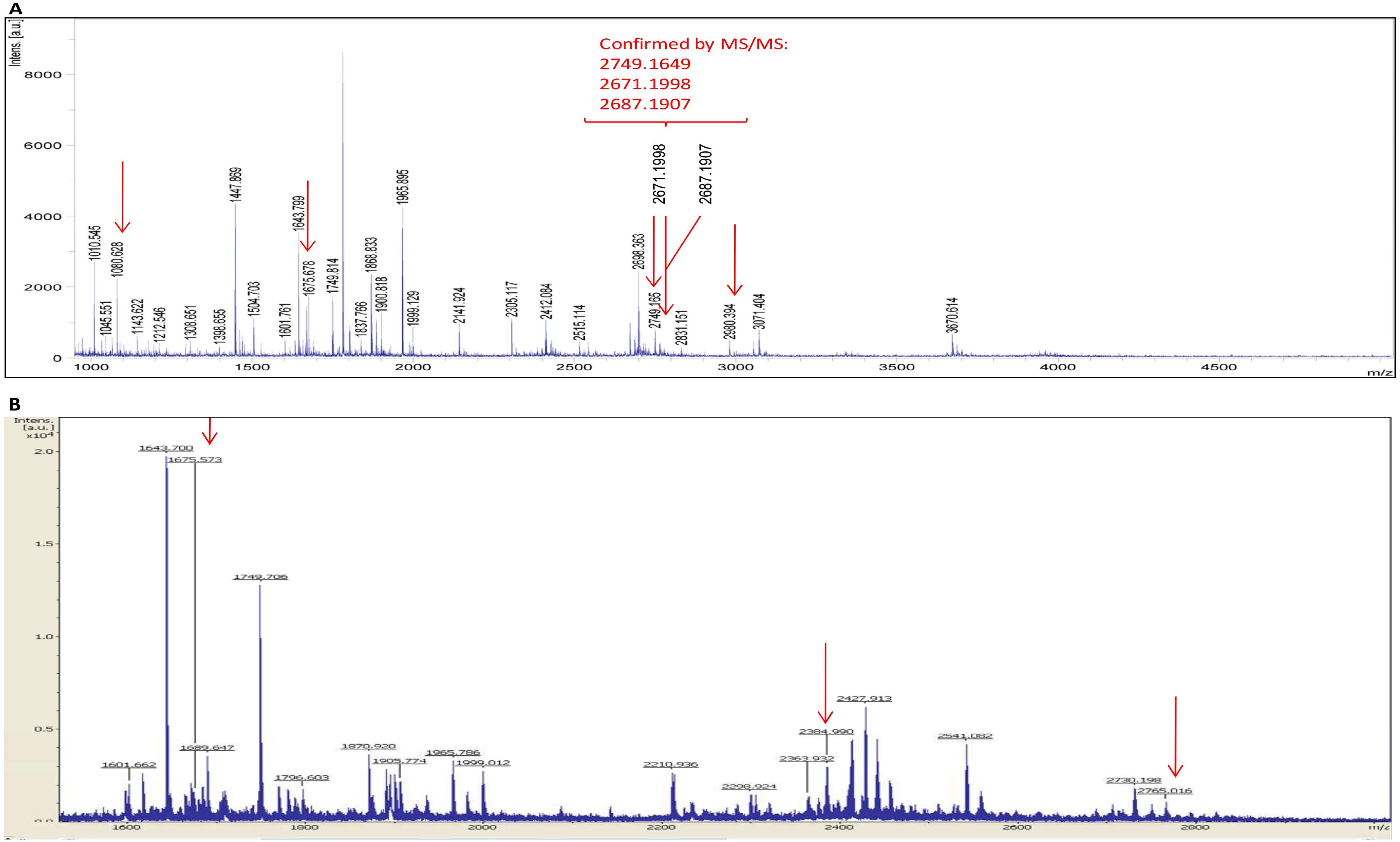

Figure 1 Maldi-ToF MS spectra obtained from an AOX1 protein spot. The red arrows indicate peptide peaks which are only present in AOX1 and not in AOX2. Three of these peptides (indicated in spectra A) were additionally submitted to MS/MS for sequence analysis.

Figures

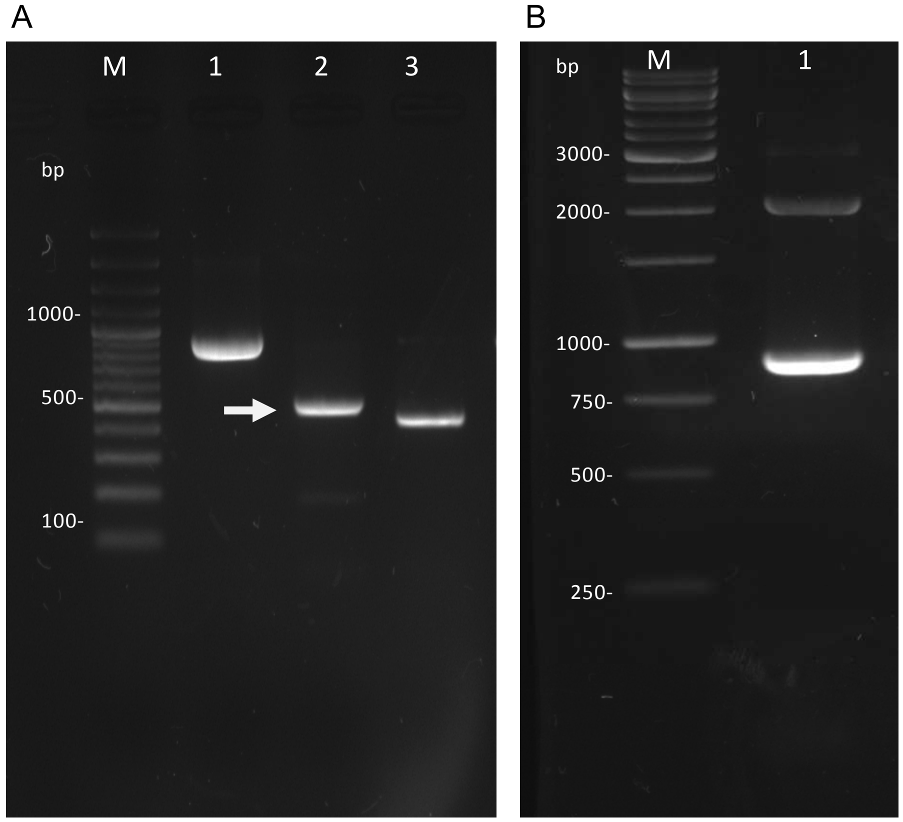

Figure 2 Agarose gel electrophoresis of PCR amplified *aox1* and *HBsAg* using *aox1* external and internal primers and *HBsAg* internal primer. (A) Represents the results for the first PCR experiment with Taq DNA polymerase and (B) the results of the second PCR experiment using the high performance polymerase, longer elongation time and higher annealing temperature. **Gel A:** M, 100bp DNA ladder (Fermentas); lane 1, *aox1* external primer (only *HBsAg* visible~900 bp), lane 2, *aox1* internal primer (*aox1* internal sequence 507 bp) and lane 3, *HBsAg* internal primer (*HBsAg* internal sequence 447pb). The band indicated by the arrow was subjected to sequencing and verified as *aox1*. **Gel B:** M, 1Kb DNA ladder (Fermentas); lane 1, with *aox1* external primer now 2 bands visible corresponding to *aox1* 2105 bp and *HBsAg* ~900bp.

Figures

```
Query  1     MAIPEEFDILVLGGGSSGSCIAGRLANLDHSLKVGLIEAGENNLNNPWVYLPGIYPRNMK 60
             MAIPEEFDILVLGGGSSGSCIAGRLANLDHSLKVGLIEAGENNLNNPWVYLPGIYPRNMK
Sbjct  1     MAIPEEFDILVLGGGSSGSCIAGRLANLDHSLKVGLIEAGENNLNNPWVYLPGIYPRNMK 60

                                         1                       2
Query  61    LDSKTASFYTSNPSPHLNGRRAIVPCANVLGGGSSINFMMYTRGSASDYDDFQAEGWKTK120
             LDSKTASFYTSNPSPHLNGRRAIVPCAN+LGGGSSINFMMYTRGSASDYDDF+AEGWKTK
Sbjct  61    LDSKTASFYTSNPSPHLNGRRAIVPCANILGGGSSINFMMYTRGSASDYDDFEAEGWKTK120

                                3                          4
Query  121   DLLPLMKKTETYQRACNNPDIHGFEGPIKVSFGNYTYPVCQDFLRASESQGIPYVDDLED180
             DLLPLMKKTETYQRACNNP+IHGFEGPIKVSFGNYTYPVCQDFLRA+ESQGIPYVDDLED
Sbjct  121   DLLPLMKKTETYQRACNNPEIHGFEGPIKVSFGNYTYPVCQDFLRATESQGIPYVDDLED180

Query  181   LVTAHGAEHWLKWINRDTGRRSDSAHAFVHSTMRNHDNLYLICNTKVDKIIVEDGRAAAV240
             LVTAHGAEHWLKWINRDTGRRSDSAHAFVHSTMRNHDNLYLICNTKVDKIIVEDGRAAAV
Sbjct  181   LVTAHGAEHWLKWINRDTGRRSDSAHAFVHSTMRNHDNLYLICNTKVDKIIVEDGRAAAV240

                      5      6    7
Query  241   RTVPSKPLNPKKPSHKIYRARKQIVLSCGTISSPLVLQRSGFGDPIKLRAAGVKPLVNLP300
             RTVPSKPLN KKP+HK+YRAR QIVLSCGTISSPLVLQRSGFGDPIKLRAAGVKPLVNLP
Sbjct  241   RTVPSKPLNAKKPTHKVYRARTQIVLSCGTISSPLVLQRSGFGDPIKLRAAGVKPLVNLP300

                                              8   9
Query  301   GVGRNFQDHYCFFSPYRIKPQYESFDDFVRGDAEIQKRVFDQWYANGTGPLATNGIEAGV360
             GVGRNFQDHYCFFSPYRIKPQYESFDDFVRGDA IQK+VFDQWYANGTGPLATNGIEAGV
Sbjct  301   GVGRNFQDHYCFFSPYRIKPQYESFDDFVRGDANIQKKVFDQWYANGTGPLATNGIEAGV360

Query  361   KIRPTPEELSQMDESFQEGYREYFEDKPDKPVMHYSIIAGFFGDHTKIPPGKYMTMFHFL420
             KIRPTPEELSQMDESFQEGYREYFEDKPDKPVMHYSIIAGFFGDHTKIPPGKYMTMFHFL
Sbjct  361   KIRPTPEELSQMDESFQEGYREYFEDKPDKPVMHYSIIAGFFGDHTKIPPGKYMTMFHFL420

                              10                  11        12
Query  421   EYPFSRGSIHITSPDPYAAPDFDPGFMNDERDMAPMVWAYKKSRETARRMDHFAGEVTSH480
             EYPFSRGSIHITSPDPYA PDFDPGFMNDERDMAPMVW+YKKSRETAR+MDHFAGEVTSH
Sbjct  421   EYPFSRGSIHITSPDPYATPDFDPGFMNDERDMAPMVWSYKKSRETARKMDHFAGEVTSH480

                        13               14              15
Query  481   HPLFPYSSEARALEMDLETSNAYGGPLNLSAGLAHGSWTQPLKKPTAKNEGHVTSNQVEL540
             HPLFPYSSEARA EMDLETSNAYGGPLNL+AGLAHGSWTQPLKKP   +NEGHVTSNQVEL
Sbjct  481   HPLFPYSSEARAYEMDLETSNAYGGPLNLTAGLAHGSWTQPLKKPAGRNEGHVTSNQVEL540

Query  541   HPDIEYDEEDDKAIENYIREHTETTWHCLGTCSIGPREGSKIVKWGGVLDHRSNVYGVKG600
             HPDIEYDEEDDKAIENYIREHTETTWHCLGTCSIGPREGSKIVKWGGVLDHRSNVYGVKG
Sbjct  541   HPDIEYDEEDDKAIENYIREHTETTWHCLGTCSIGPREGSKIVKWGGVLDHRSNVYGVKG600

                                                  16
Query  601   LKVGDLSVCPDNVGCNTYTTALLIGEKTATLVGEDLGYSGEALDMTVPQFKLGTYEKTGL660
             LKVGDLSVCPDNVGCNTYTTALLIGEKTATLVGEDLGY+GEALDMTVPQFKLGTYEKTGL
Sbjct  601   LKVGDLSVCPDNVGCNTYTTALLIGEKTATLVGEDLGYTGEALDMTVPQFKLGTYEKTGL660

Query  661   ARF   663
             ARF
Sbjct  661   ARF   663
```

Identities = 644/663 (98%), positives = 656/663 (99%), gaps = 0/663 (0%) Query 1= AOX1 Sbjct 1= AOX2

Figure 3 Sequence differences between AOX1 and AOX2. Amino acid sequences of AOX1 and AOX2 are from the Uniprot database (accession number Q9URI8 and Q9URI7, respectively). Protein homologies were determined using NCBI BLAST program via NCBI web-server. Amino acids with green background represent the differences between AOX1 and AOX2.

References

1. Vassileva A, Chugh DA, Swaminathan S, Khanna N: **Effect of copy number on the expression levels of hepatitis B surface antigen in the methylotrophic yeast *Pichia pastoris*.** *Protein Expr Purif* 2001, **21:** 71-80.

2. Zhu T, Guo M, Tang Z, Zhang M, Zhuang Y, Chu J *et al.*: **Efficient generation of multi-copy strains for optimizing secretory expression of porcine insulin precursor in yeast *Pichia pastoris*.** *J Appl Microbiol* 2009, **107:** 954-963.

3. Gurramkonda C, Adnan A, Gäbel T, Lünsdorf H, Ross A, Nemani SK *et al.*: **Simple high-cell density fed-batch technique for high-level recombinant protein production with *Pichia pastoris*: Application to intracellular production of Hepatitis B surface antigen.** *Microb Cell Fact* 2009, **8:** 13.

4. Lünsdorf H, Gurramkonda C, Adnan A, Khanna N, Rinas U: **Virus-like particle production with yeast: ultrastructural and immunocytochemical insights into *Pichia pastoris* producing high levels of the Hepatitis B surface antigen.** *Microb Cell Fact* 2011, **10:** 48.
